# Detecting protein folding by thermal fluctuations of microcantilevers

**DOI:** 10.1371/journal.pone.0189979

**Published:** 2017-12-21

**Authors:** Romina Muñoz, Felipe Aguilar-Sandoval, Ludovic Bellon, Francisco Melo

**Affiliations:** 1 Departamento de Física, Facultad de Ciencia, Universidad de Santiago de Chile, Santiago, Chile; 2 Departamento de Física, Facultad de Ciencias Físicas y Matemáticas, Universidad de Chile, Santiago, Chile; 3 Laboratoire de Physique, ENS de Lyon, Univ Claude Bernard Lyon 1, Univ Lyon, CNRS, Lyon, France; National Institutes of Health, UNITED STATES

## Abstract

The accurate characterization of proteins in both their native and denatured states is essential to effectively understand protein function, folding and stability. As a proof of concept, a micro rheological method is applied, based on the characterization of thermal fluctuations of a micro cantilever immersed in a bovine serum albumin solution, to assess changes in the viscosity associated with modifications in the protein’s structure under the denaturant effect of urea. Through modeling the power spectrum density of the cantilever’s fluctuations over a broad frequency band, it is possible to implement a fitting procedure to accurately determine the viscosity of the fluid, even at low volumes. Increases in viscosity during the denaturant process are identified using the assumption that the protein is a hard sphere, with a hydrodynamic radius that increases during unfolding. This is modeled accordingly through the Einstein-Batchelor formula. The Einstein-Batchelor formula estimates are verified through dynamic light scattering, which measures the hydrodynamic radius of proteins. Thus, this methodology is proven to be suitable for the study of protein folding in samples of small size at vanishing shear stresses.

## Introduction

Rheological measurements are recognized as suitable for in the assessment of unfolding phenomena [1], as the hydrodynamic volume of a protein at sufficiently high concentrations can have a significant impact on the viscosity of a protein solution. However classical rheometers, that apply relatively high shearing, can promote artificial losses in protein structure, resulting in biased measurements. These effects have been found to be more prominent in proteins with high content of alpha helix domains [2, 3] whereas they are dramatically reduced for the cytochrome c protein [4]. In addition, due to their principle of functioning, classical rheometers require large sample volumes at sufficiently high protein concentrations, which may increase the cost of an assay and make it difficult to detect minute variations in viscosity in small samples. Thus, classical rheological methods to investigate protein folding are currently only used to verify results. However, a wide variety of modern rheometers, such as micro fluidity capillary rheometers, have been developed to push down the limit of detection to samples less than 100 *μ*l with good accuracy [5].

Recently, it was shown that microrheology methods are able to overcome these previously mentioned shortcomings. Indeed, passive rheology has emerged as a suitable methodology to decrease sample size to a few micro liters, to increase the frequency response and to reduce the shear rates applied to proteins [6].

Passive rheology is achieved through the monitoring of the Brownian trajectories of colloidal probe particles (BTCP) within microliter-sized samples [6]. Detection of viscosity changes in a protein solution is demonstrated through the addition of a denaturant that induces an increase in the protein size during unfolding. Globular proteins were treated as hard-spheres and the viscosity modeled according to the diluted-suspensions theory first developed by Einstein and Batchelor. Viscosity was directly obtained by measuring how colloidal particles diffuse in the protein solution and by using well known Einstein’s formulas of Brownian motion [6]. The hydrodynamic radius (*R*_*H*_) of the protein in the folded and unfolded states was determined by adjusting the measured viscosity values to Batchelor’s expression. However, simultaneous and independent measurements of the protein size were not taken, thus it was not possible to compare results.

Previously, our group developed a suitable device that can sense thermal vibrations of a cantilever immersed in a minute amount of testing fluid [7]. This enabled further exploration of cantilevers as a potential viscosity sensor, suitable in situations with reduced testing fluid. Indeed, an interferometric atomic force sensor that was previously used for gas measurements [8], was refined to measure fluid viscosity, enabling the analysis of the power spectrum density (PSD) of the fluctuating cantilever-deflection, and thus rendering the sensor suitable for use with liquids. This methodology used a full fit of the PSD, including the first two vibration modes of the cantilever, through the mathematical expression provided by Saders’s model [9]. By contrast, previous investigations were either based on using the fit of PSD in a limited frequency band [10], or the shift in the resonance frequency of cantilever with viscosity [11]. Although the full fit method of PSD is more laborious in terms of calculations, it improves the resolution for viscosity determination decreasing uncertainties down to 1%.

In this article, the capacity of micro cantilevers to assess protein folding is investigated. Bovine serum albumin (BSA) is selected as a model globular protein because it has a well-known unfolding transition under the action of denaturant urea [12]. Protein solution viscosity is determined through the analysis of the thermal fluctuations of a soft micro cantilever, whose PSD conforms to Sader’s model. The protein *R*_*H*_ is then determined through the Einstein-Batchelor formula.

There is evidence indicating that under the effect of denaturant urea, the dynamic light scattering technique (DLS) is useful to determine the *R*_*H*_ of BSA [13]. In this work, under similar experimental conditions including denaturant action, DLS allows for independent measurements to be taken for the *R*_*H*_ protein average, providing basis for comparing both techniques.

Under the folded protein state, both methodologies give consistent results for *R*_*H*_ values, as a function of denaturant concentration, which validates the use of the Einstein-Batchelor formula to describe variations in protein size within suspensions. When the denaturant reaches mid-concentrations, at which point the transition to the unfolded state occurs, DLS has a tendency to underestimate protein *R*_*H*_. This may be due to the gradual coexistence of two distinct protein populations, with both folded and unfolded proteins.

## Physical background

### Cantilever response to thermal fluctuations

In summary, the main physical mechanism influencing cantilever response to thermal fluctuation, and its latter application to sensing fluid viscosity, can be shown through a single harmonic oscillator (SHO) approximation. A more detailed description, with references, is available in the S1 File. The SHO is driven by thermal fluctuations and described by a Langevin’s type equation,

$$\begin{array}{c} m_{\text{eff}}\ddot{d}+\gamma_{\text{eff}}\dot{d}+kd=F_{T}\left(t\right) \end{array}$$ (1)

where *d* is the cantilever deflection, *m*_eff_ is the total effective mass accounting for the inertia of the cantilever and an added equivalent mass due to the motion of the surrounding fluid, *γ*_eff_ is the effective viscous damping around the cantilever and *k* is the cantilever’s stiffness. *F*_*T*_(*t*) is the random force due to thermal fluctuations characterized by time average, < *F*_*T*_ > = 0, and autocorrelation, < *F*_*T*_(*t*)*F*_*T*_(*t* − *τ*) > = 2*γ*_eff_ *k*_*B*_ *Tδ*(*τ*), where *k*_*B*_ *T* is the thermal energy and *δ*(*τ*) the Dirac *δ* function. Solving Eq 1 in the frequency domain gives *d*(*ω*) = (*k* − *m*_eff_ *ω*^2^ + *iγ*_eff_ *ω*)^−1^ *F*_*T*_(*ω*). The PSD, *S*_*d*_(*ω*), of cantilever deflection is defined as,

$$\begin{array}{c} S_{d}\left(\omega \right)=\lim_{t_{s}\to \infty }\left(\frac{d\left(\omega \right)d^{*}\left(\omega \right)}{\pi t_{s}}\right), \end{array}$$ (2)

where *t*_*s*_ is the sampling interval. Note that with this definition $<d^{2}>=\int_{0}^{\infty }S_{d}\left(\omega \right)\;d\omega$. According to the Wiener-Khinchin theorem, the PSD of the fluctuating force is simply 2*γ*_eff_ *k*_*B*_ *T*, which ultimately results in the expression of the fluctuation dissipation theorem for a SHO as,

$$\begin{array}{c} S_{d}\left(\omega \right)=\frac{2k_{B}T}{\pi }\frac{\gamma_{\text{eff}}\left(\omega \right)}{{\left(k-m_{\text{eff}}\left(\omega \right)\omega^{2}\right)}^{2}+{\left(\gamma_{\text{eff}}\left(\omega \right)\omega \right)}^{2}} \end{array}$$ (3)

Through the theoretical expressions of *γ*_eff_ and *m*_eff_, in terms of the hydrodynamical function Γ[9], given in the S1 File, the vacuum resonance frequency, *ω*_0_, relates to the resonance frequency, *ω*_*r*_, [9] according to,

$$\begin{array}{c} \omega_{0}=\omega_{r}\sqrt{1+\frac{\pi \rho W}{4\rho_{0}e}\Gamma_{r}\left(\omega_{r}\right)} \end{array}$$ (4)

Where *W* and *e* are the cantilever’s width and thickness, respectively; *ρ* and *ρ*_0_ are the fluid and cantilever density, respectively. It was observed that, *ω*_*r*_ is strictly defined as the frequency at which the real part of the response function is zero, e.g. when $m_{eff}\left(\omega_{r}\right)\omega_{r}^{2}=k$. The mathematical expression for Γ_*r*_, the real part of Γ, involves Bessel functions which are difficult to manipulate. Therefore, in order to describe the influence of fluid motion on the oscillating behavior of the cantilever, the analytical approximation given in [14] is used, written as,

$$\begin{array}{c} \Gamma_{r}\left(\omega_{r}\right)=a_{1}+a_{2}\frac{1}{W}\sqrt{\frac{2\eta }{\rho \omega_{r}}}, \end{array}$$ (5)

where *a*_1_ = 1.0553 and *a*_2_ = 3.7997. Finally, through Eqs 4 and 5, the fluid viscosity *η*, in terms of the resonance frequency writes,

$$\begin{array}{c} \eta =c_{1}\cdot \omega_{r}{\left[\frac{\omega_{0}^{2}-\omega_{r}^{2}}{\omega_{r}^{2}}-c_{2}\right]}^{2} \end{array}$$ (6)

where $c_{1}=\frac{8}{\pi^{2}}\frac{\rho_{0}^{2}}{\rho }\frac{e^{2}}{a_{2}^{2}}$ and $c_{2}=\frac{\pi }{4}\frac{\rho }{\rho_{0}}\frac{W}{e}a_{1}$. Eq 6 explains how the PSD of fluctuations are affected by dissipation changes: in fact the PSD maximum moves to lower frequencies with increasing viscosity, which is an indication of increasing inertia, since there is greater fluid resistance during cantilever motion. This work shows that Eq 6 can be used to rapidly estimate the viscosity of a protein solution, through monitoring the resonant frequency *ω*_*r*_. In addition, Einstein-Batchelor’s formula may be used to estimate the average *R*_*H*_ of a protein.

Similar methodologies, based on the application of Sader’s model and the optical lever method, can be used in the analysis to detect deflections and determine fluid viscosity [15][10, 11, 16, 17]. However, in general, fluid viscosity is obtained through the partial fit of the PSD spectra (e.g. from the first resonance) [17], or by solving an approximated equation that links both the frequency shift (e.g. Eq 6) and the quality factor to the fluid viscosity and density [10, 11], with an approximately 5% accuracy. Hence, in order to improve accuracy, a full fit of the PSD is carried out in Eq 3, with a simultaneous accurate description of the cantilever’s mechanical and geometrical properties. This protocol requires the fine tuning of the geometrical features of the cantilever, using a reference fluid with a known viscosity and density. It also involves efficient fitting procedures, which are described in detail in the S1 File. To conclude this section let us mention that the frequency response of a damped oscillator was used early to characterize fluid rheology near a critical point [18]. The in-phase and out of phase components of the oscillator response allowed for the assessment of the viscosity and viscoelastic contributions through a calibration procedure aiming at characterizing the damping term.

### Vanishing stress rheology

Applying a substantial shear stress to a protein may significantly affect its natural conformation state. There are several studies characterizing the secondary and tertiary structure of proteins immediately after proteins are subjected to high “shear flow” without finding any evidence of changes in protein structure, as demonstrated by Jaspe et al. [4] in the cytochrome c protein. However, subsequent works demonstrate the susceptibility of some proteins to undergo structural changes under shear. For instance, it was shown through the Raman spectroscopy technique that proteins with high content of alpha helical structure are significantly affected in their tertiary structure after being exposed to shear stress [2] and that a relationship between the alpha helix content of the protein and its ability to undergo conformational changes under flow exists. Moreover, Bekard et al [3] demonstrated through fluorescence and circular dichroism spectroscopy that a “simple shear flow is capable of disrupting the tertiary structure and unfolding the *α*-helical segments of a natively folded BSA”: the BSA tertiary configuration and helical segments dissociate/unfold at shear rates of 45 s^−1^ and 100 s^−1^ respectively. Notice that these values are smaller than the typical shear rate used in conventional rheometers.

Therefore, when these methods are used to characterize proteins, it is important to know the typical shear strain, *γ* and shear rate, $\dot{\gamma }$, imposed upon the sample due to cantilever thermal fluctuations. Indeed, from energy equipartition, *k* < *d*^2^ > = *k*_*B*_*T*, for a soft cantilever with a stiffness of 0.03 N/m, at room temperature the root-mean-square displacement of thermal motion is about 0.4 nm, and the associated cantilever speed is around $\omega \sqrt{<d^{2}>}$. Thus, $\dot{\gamma }\approx \omega \sqrt{<d^{2}>}/\delta$, where $\delta =\sqrt{2\eta /\rho \omega }$ is the penetration length of the flow. In water, *f* = 3*kHz* (considering the typical resonance frequency of a cantilever) this is *δ* ≈ 0.1 *μ*m, *γ* ≈ 10^−4^ and $\dot{\gamma }\approx 1\;{\text{s}}^{\text{-1}}$. Thus, the maximum force, *F*, acting on a single protein within the shear region, writes as, $F\approx \eta \dot{\gamma }D^{2}$, with *D* being the standard protein size, *D* ≈ 10 nm. The result is *F* < 10^−19^ N, which is several orders of magnitude smaller than the force required to unfold a protein, as demonstrated by single molecules assays [19].

### Viscosity of a colloidal suspension: Einstein-Batchelor’s formula

Batchelor’s improvement of Einstein’s model predicts that the relative viscosity of a suspension of hard spheres will vary as a quadratic function of volume fraction, with the following coefficients,

$$\begin{array}{c} \eta =\eta_{s}\cdot \left(1+2.5\phi +6.2\phi^{2}\right) \end{array}$$ (7)

where *ϕ* is the equivalent spherical volume fraction occupied by the protein, and *η* and *η*_*s*_ are the measured viscosity and the solute-free solvent viscosity, respectively. Batchelor’s contribution accounts for both the two-body hydrodynamics interactions and the Brownian contribution [20], thus providing a work frame for the analysis at higher protein concentrations. However, for volume fractions greater than 20% and in the presence of prominent interparticle interaction, Batchelor’s expression begins to underestimate the relative viscosity [21].

The unfolding of the protein under the effect of the denaturant and the increase in the BSA concentration, are two factors that increase the volume fraction occupied by the protein. This increase in *ϕ* is reflected as a change in the viscosity of the solution, as predicted by the Eq 7. Notice that the volume fraction links to the equivalent protein radius *R*_*H*_ and concentration, *c*, as $\phi =c\cdot N_{a}\frac{4}{3}\pi R_{H}^{3}$, where *c* is expressed in mM, and *N*_*a*_ is the Avogadro’s number.

Considering the hydration layer of the protein is important to estimate correctly the protein size [22]. Notice that the two main contributions to the protein volume, namely the dry-bare protein volume and the hydration layer volume are included in the equivalent protein radius. An alternative description considering these two contributions separately in the Eq 7 is presented in the work of Sarangapani et al. [23].

## Materials and methods

To carry out the *R*_*H*_ assessment of the unfolded protein, eight different stock solutions of BSA (from Sigma A2153) were prepared by dissolving the crystalline protein, at an initial concentration of 0.6 mM, in eight different concentrations of urea (Sigma U5378), ranging from 0 to 7M, respectively. Urea solutions are prepared in 100 mM of sodium phosphate buffer at pH 7.2 and 150 mM of NaCl. Each stock solution was progressively diluted to obtain seven samples of BSA, with concentrations ranging from 0 to 0.6mM, and with equal concentrations of urea (from 0 to 7 M). This procedure generates 56 different samples for viscosity testing. All samples were incubated for 24 Hrs. The concentration of BSA was determined by measuring the maximum of absorbance at 280 (nm). To determine the molar concentration, the molar extinction coefficient for BSA of 43.824 (M cm)^−1^ was used [24]. The conversion from mass to moles has already been considered by means of the molar mass of BSA, 66400 g/mol; the molecular weight of the BSA monomer.

### Cantilever thermal fluctuations

**Fig 1:**
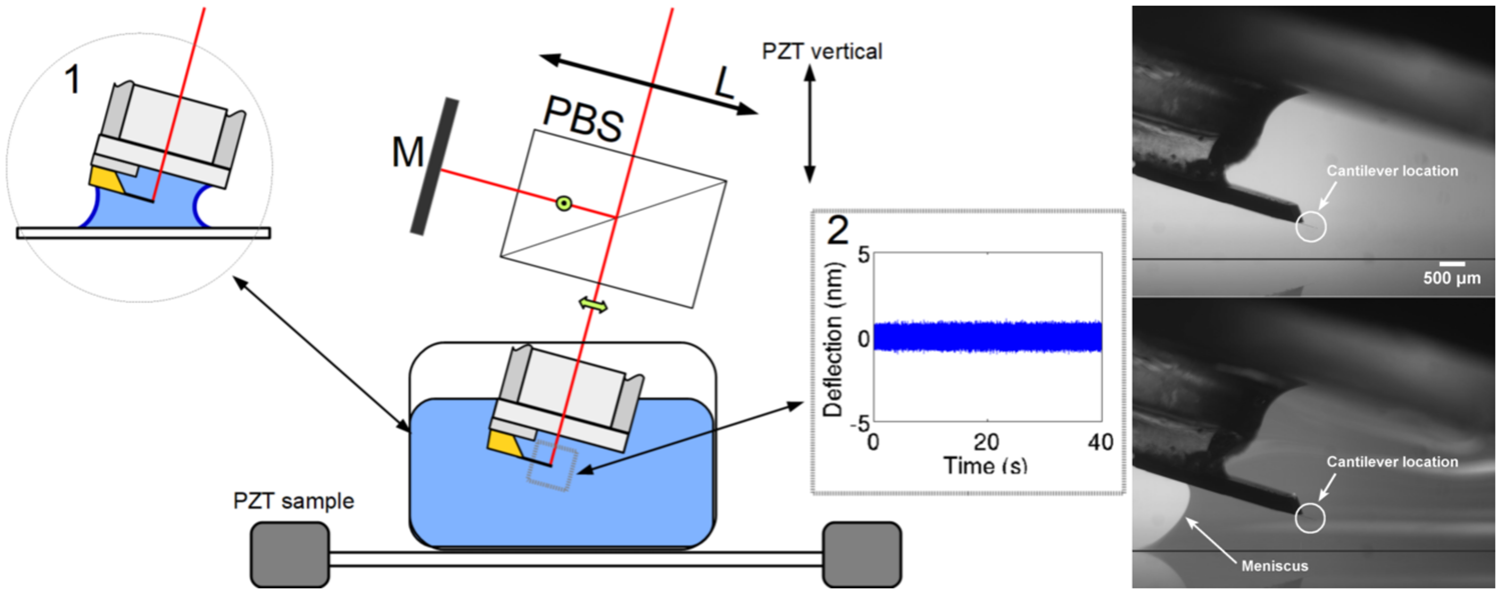
Experimental setup. A) Schema of the experimental configuration for detecting deflections of a cantilever immersed in a protein solution. A polarizing beam splitter (PBS) divides the input into two beams of crossed polarizations. A mirror (M) reflects the reference beam and provides the overlap of the returned beams. A lens (L) focuses the probe beam at the free end of the cantilever. The reflected beams, probe and reference, interfere after projecting the initial polarizations in the analysis area (see [8] for details). The standard immersion is achieved in a 1.5 ml fluid cell. Inset 1: The meniscus configuration uses a volume smaller than 50 *μ*l. Inset 2: Typical time trace of deflection fluctuations. B) Side view of the cantilever with and without the fluid meniscus.

Viscosity assessment was based on detecting fluctuations of the micro cantilever induced by the thermal energy of the bath. As previously described, the PSD of fluctuations are influenced by the rheological properties of the aqueous solution, primarily density and viscosity, where the cantilever is immersed. A system using a quadrature phase interferometer is used to detect deflections of the micro cantilever. A detailed description of this device is provided by [8], and represented schematically in Fig 1A, with a brief description. In this case, the setup includes a reference beam located outside of the fluid cell using a Michelson-like polarized configuration. An external mirror (M) reflects the reference beam and provides fine control of any overlap in the returned beams. Accurate positioning of the lens (L) is controlled through a motorized three-axis system to focus the probe beam (z-control), and facilitates the accurate positioning of the laser spot at the free end of the cantilever. To accurately model cantilever fluctuations, it is important to know the exact position of the laser spot. During experimentation, a silicon made AFM cantilever tip-less probe is used *HQ:CSC38* (from mikromasch) with stiffness *k* = 0.03 N/m, and a rectangular shape, *L* = 350 *μ*m, *W* = 32.5 *μ*m and *e* = 1 *μ*m.

The basic setup includes a cylindrical fluid cell made of glass with a volume capacity of 1.5 ml, to enable full immersion of the cantilever and its holder into the testing fluid. The inset 1 in Fig 1A indicates a second configuration, where a small meniscus is formed between the cantilever holder and a horizontal coverslip, located approximately 2 mm directly below the cantilever. A stable meniscus is achieved through the gradual addition about 50 *μ*l of aqueous solution through a micropipette located near to the base of the cantilever. Meniscus evaporation is minimized and no significant mass variation occurred during the first and last runs. This configuration allows for the testing of small quantities of fluids, approximately 50 *μ*l, without any loss of sensitivity. Notice that the cantilever is at a distance of about a millimeter from the air/solution interface and is totally immersed into the solution, which prevents any additional viscous effect due to stiffening associated with the adsorption of BSA at this interface [25].

Acquisition of cantilever deflection is carried out at a sampling rate of 1 MHz for 2 s, at 16 bits resolution. Inset 2 in Fig 1A presents results from 40 s of signal. Acquisition is repeated 20 times for each sample in order to obtain a good PSD average. Thus, under these conditions, the time resolution of our methodology is limited to 40 s, and may be reduced to 1 s or even lower, however it will lead to some decreased accuracy (see Discussion).

The direct signal from the deflection is then processed in order to obtain the PSD, and thus reveal the viscosity of the surrounding fluid. Provided that the geometrical parameters of the cantilevers are known, a fitting procedure can be used, considering the mathematical expression of the PSD of the cantilever deflections, to determine the fluid properties. The S1 File includes a summary of the main steps, and a detailed protocol, including the mathematical aspects of the viscosity assessment.

**Fig 2:**
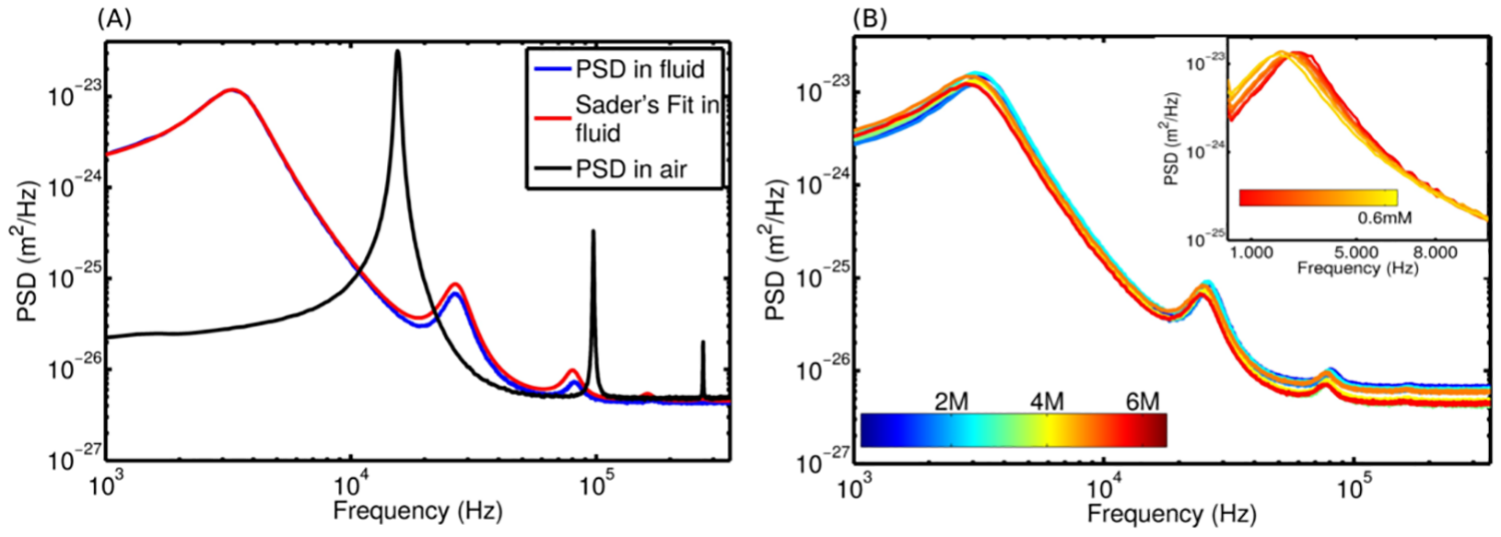
Power spectrum density of cantilever’s deflections. A) PSDs in air and water with their respective Sader’s model fit in fluid. B) PSDs of several mixtures of urea, the color bar represents the urea concentration. The inset in B shows the PSDs for different BSA concentrations, at a constant urea concentration of 7M. The color bar in the inset figure represents the BSA concentration from 0 to 0.6 mM.

Despite the fact that the cantilever has well-established geometrical properties, including width, length, thickness and stiffness, the accuracy of this method could be further improved through a fine tuning of these parameters. This is due to the imperfect shape of cantilevers, which creates uncertainties in the physical parameters of the cantilever. Primarily, these geometrical parameters are determined by means of an inverse fitting procedure, as follows:

- the cantilever is immersed in ultra-pure water, with a known density and viscosity at a given temperature. The PSDs are then registered and the geometrical parameters, *L*, *W*, *e* and *k*, are adjusted so that optimum agreement is achieved with the experimental PSD.
- A similar procedure is followed to determine an unknown fluid viscosity, however the previously determined cantilever’s geometry and stiffness are maintained while the viscosity and density are free parameters within the fitting procedure. Typical experimental PSDs and their corresponding Sader’s Fits in air and water are shown in Fig 2A. A change in the viscosity of the media is reflected by a change in the PSD of the cantilever (Fig 2B), and further adjustments to Sader’s model reveals the new viscosity value.

When possible, it is recommended to carry out a rapid and qualitative check of the *R*_*H*_ of a protein solution. For such situations, the previous methodology can be simplified by determining the *η* through the direct measurement of the resonance frequency *ω*_*r*_, using Eq 6. Indeed *ω*_*r*_ can be directly obtained through the local fit of the *S*_*d*_ maximum, using the gaussian function. The resulting viscosity values can be inserted into the Einstein-Batchelor’s expression, Eq 7, which gives the average of the hydrodynamic radius of the protein. However, this simplification leads to a greater uncertainty in viscosity and *R*_*H*_ compared to the full fit from Sader’s model.

### Dynamic light scattering

To determine the distribution of the protein *R*_*H*_ through the DLS technique, the Malvern Zetasizer Nano ZS (Malvern Instruments, UK) was used. All samples were measured in triplicate. To determine the hydrodynamic size of a given sample, the signal from DLS was integrated during 15 s, and repeated 15 times, in order to produce a representative distribution. The data obtained were exported at optimum best resolution using the Zetasizer program, obtaining a resolution of 0.083 nm in radius. The intensity distribution was used in all measurements, since this distribution only requires the identification of solvent parameters, opposed to number and volume distributions.

The samples are prepared with a constant BSA concentration of 0.2 mM, and with various urea concentrations ranging from 0 to 7 M. A 500 *μ*l solution volume was used for all DLS measurements.

It should be considered that during the analysis of DLS data, the solution viscosity varies depending on the denaturant concentration. This is simple and straightforward to correct using the Zetasizer software; by replacing the dispersant with a mixture of urea and water, available in the complex material options. Thus, all the radius distribution measurements in this study are adjusted to the actual solvent viscosity.

It is well known that the concentration dependence of the apparent diffusion coefficient gives information about the protein interactions. The effect of the protein concentration is considered by extrapolating the value of the apparent diffusion coefficient, *D*_*app*_ to that at zero concentration, *D*_0_, as described in [23]. A linear fit of *D*_*app*_ versus *c*_*BSA*_, with *D*_*app*_ = *D*_0_(1+*k*_*D*_ *c*_*BSA*_) is used to obtain the diffusion interaction parameter, *k*_*D*_, which accounts for intermolecular thermodynamic and hydrodynamic contributions (see S1 File and S5 Fig).

## Results

**Fig 3:**
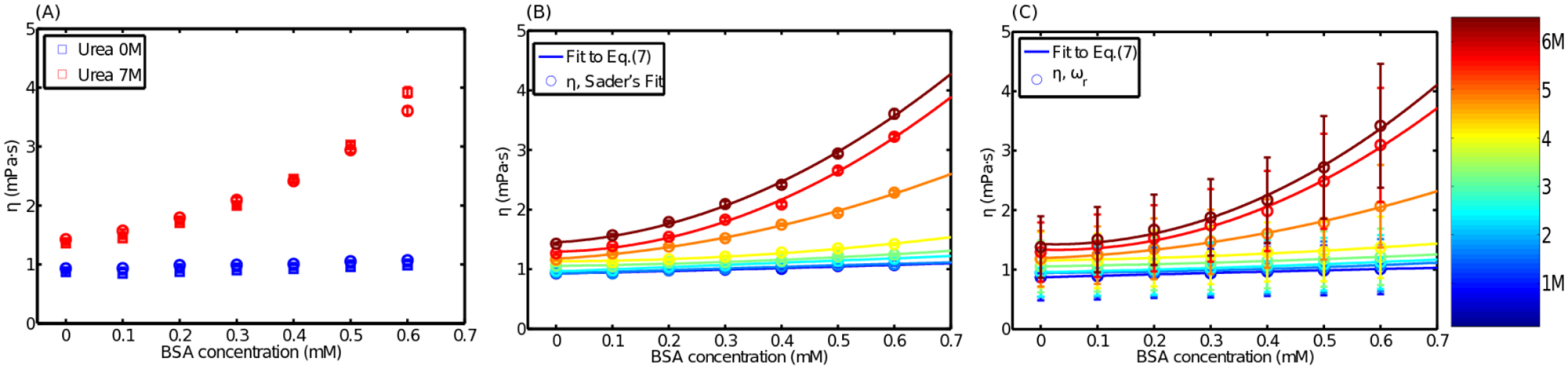
Viscosity of the folded and unfolded protein. A) Bulk viscosity variations with increasing concentration of BSA (square markers): in a pure buffer (blue markers), and a buffer with 7 M of urea (red markers). These measurements are repeated for 50 *μ*L volume in the meniscus configuration (circles markers). The experiments were carried out at *T* = 25°C and *T* = 26°C in the meniscus and the conventional configurations, respectively. B) The full data set for several urea concentrations are fitted to Eq 7 using the hydrodynamic radius as adjustable parameter. C) A full set of solution viscosities is obtained from direct measurements of *ω*_*r*_ (Eq 6) through a Gaussian fit to the maximum PSD. Symbols represent the experimental data and lines represent the corresponding fit. The color scale, at the right hand side of panel C, represents the urea concentration in panels B and C. Note that $\phi =c\cdot N_{a}\frac{4}{3}\pi R_{H}^{3}$, with *c* in mM.

Primarily, this study has demonstrated the potential to measure protein solution viscosity at very low sample volumes, through the fitting of the observed PSD to its theoretical expression in both volume configurations. Considering these results, the effect that the denaturant concentration has on the viscosity of the BSA-denaturant solution is further explored; specifically under conditions of increasing BSA concentrations and for two distinct urea concentrations (Fig 3A). The first data set, obtained with zero urea concentration (blue data), shows minimal variation in solution viscosity with BSA concentration, indicating that the protein remained in a folded state, regardless of BSA concentration. However, viscosity shows a linear dependency on BSA concentration, which is characteristic of low *ϕ*. By contrast, at urea concentration of 7M, solution viscosity increases with BSA concentration. This is consistent with the behavior of BSA protein in an unfolded state, and with the quadratic dependence on *ϕ* predicted by the Eq 7. The above steps are detectable and repeatable, regardless of the sample volume. This is demonstrated by the superposition of data obtained through both methods; the conventional immersion method and the meniscus mode method (Fig 3A). Thus, considering the superposition of the data, the meniscus configuration mode is validated. However, a careful manipulation of the fluid solution is required to form the meniscus; therefore in the following measurements, the method is only used in the conventional immersion configuration.

**Fig 4:**
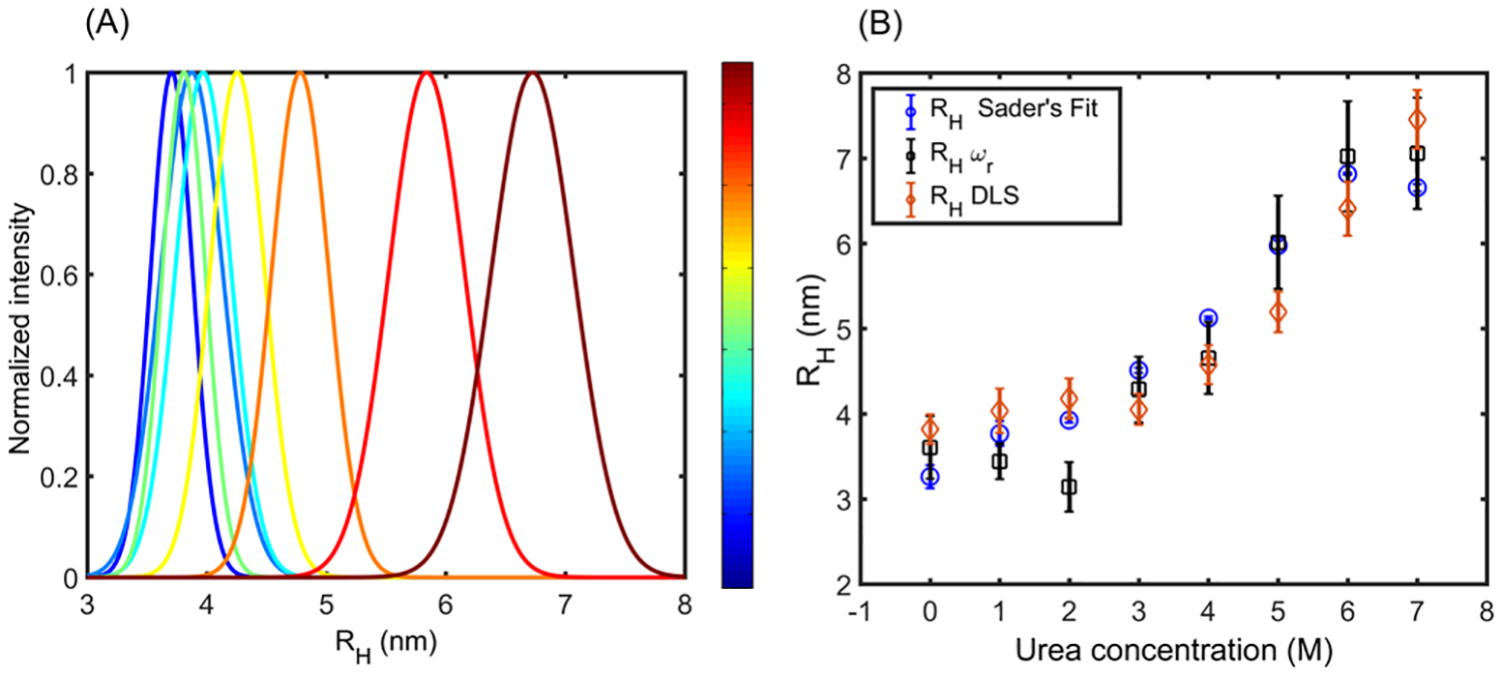
Probability distributions of hydrodynamic radius obtained by DLS. A) Distribution of protein radius for 0.2 mM BSA and distinct values of urea concentrations. Color bar represents urea concentration. Distributions indicate progressive protein unfolding with increasing urea concentration. B) Comparisons of hydrodynamic radius, obtained from fits based in Eq 6 and full Sader’s model (Fig 3B), with values from DLS distributions (Fig 4A).

A full set of viscosity data for variable concentrations of both BSA and denaturant is shown in Fig 3B. When the urea concentrations reach values lower than 4 M, the curves nearly collapse, reflecting the well-known behavior under conditions of BSA unfolding. By contrast, curves spread under higher denaturant concentrations, indicating an increase in the hydrodynamic radius. For each given value of the denaturant concentration, the experimental curve can be accurately fitted to the Eq 7 (Fig 3B) using the volume fraction as a free parameter. Since the number of BSA molecules per unit of volume is known, the value of the hydrodynamic radius *R*_*H*_ as a function of denaturant is directly obtained as $\phi =c\cdot N_{a}\frac{4}{3}\pi R_{H}^{3}$ (with *c* in mM units). For the protein folded state, *R*_*Hf*_ = 3.3± 0.1 nm is obtained, whereas for the unfolded state *R*_*Huf*_ = 6.7± 0.1 nm is obtained. The entire dataset for the hydrodynamic radius are presented in Fig 4B, as a function of urea concentration, enabling further comparison with DLS data. In addition, Table 1 summarizes *R*_*H*_ data provided by previous studies, for further comparison with these results.

**Table 1:** Hydrodynamic radius data of folded and unfolded protein. Data reported from previous studies is compared with the present results.

Reference	*R*_*H*_ folded	*R*_*H*_ unfolded
Willkins 1999	3.0 ± 0.7	8.3 ± 4.0
Tu 2005	3.4 ± 0.1	7.5 ± 0.1
Adele 2007	3.8 ± 0.2	8.3 ± 0.5
**This work**		
Sader’s Fit	3.3 ± 0.1	6.7 ± 0.1
DLS	3.7 ± 0.2	6.7 ± 0.3
*ω*_*r*_	3.6 ± 0.4	7.1 ± 0.7

A full set of solution viscosities obtained from direct measurements of *ω*_*r*_ (Eq 6), through a Gaussian fit to the PSD maximum, is given in Fig 3C. Despite some considerable uncertainties in these data in comparison with Fig 3B, they are useful for making rapid estimates of hydrodynamic radius through the fit provided by Eq 7. It is noted that Eq 6 requires the careful adjustment of parameters *c*_1_ and *c*_2_, achieved using known water viscosity values. A description of this procedure, which is based on [11], and an explanation of uncertainties in estimations for *η* and *R*_*H*_, can be found in the S1 File.

The Fig 4A shows the normalized DLS intensity distributions for constant BSA concentration and increasing urea concentration. These data reflect the progressive protein unfolding under the action of urea through the increase in the mean value of these distributions. The hydrodynamic radius provided by DLS are then contrasted with the corresponding protein radius, obtained via the viscosity assessment (Fig 4B). Results are considered from both Sader’s model, including the full fit of PSD, and from resonance frequency. Over the entire range of urea concentration, these techniques provide values of hydrodynamic radius that are in good agreement within the confident interval. Overall, these results from Sader’s model, covering the entire urea concentration range, are in close agreement with those reported by Tu [6] using the BTCP methodology, under similar conditions. Moreover, it is observed that with the approximated procedure (fit of *ω*_*r*_) results closely follow those provided by Sader’s fit, although there are greater uncertainties.

## Discussion

Despite some relative mathematical complexity, this methodology provides a rapid, non-intrusive technique to obtain viscosity and average values of the hydrodynamic radius of a protein in sample solutions of reduced volume. Both methodologies used for the cantilever fluctuation analysis give similar mean values for *η* and protein *R*_*H*_, which also concur with the expected values for the nominal solvent viscosity (see S1 File). Sader’s fit provides relative errors of about 1%, for both *R*_*H*_ and *η*, however viscosity calculations through shifting *ω*_*r*_ gives larger uncertainties; of about 30% and 15% for *η* and *R*_*H*_, respectively. Despite these errors, the latter is mathematically simpler to implement. In both cases, the mean values for *R*_*H*_ are compatible with those obtained from the validation technique (DLS), within 10%, under similar conditions for the both folded and unfolded states. However, at intermediate urea concentrations, DLS provides lower values in a range of 20%.

Herein, the advantages and disadvantages of this cantilever fluctuation methodology are compared with another approach based on monitoring the Brownian trajectories of colloidal probe particles (BTCP) embedded in microliter-sized samples [6]. Both methods share the advantage of being able to sense “local” viscosity, either within the cantilever effective volume or within a volume where Brownian fluctuations of the colloidal particles are explored. In both cases this volume is less than 1*μ*l. Concerning the protein’s dynamic assessment, this methodology improves the time resolution by up to 1 point/s, allowing relatively slow unfolding protein kinetics to be captured. For example, the unfolding time with disulfide ranges from 0.1 s to 1 min, depending of the protein and the respective disulfide bond [26]. As a proof of concept, this study successfully followed the process of slow kinetic disulfide unfolding of a BSA protein, under the action of guanidine hydrochloride plus 2-mercaptoetanol.

Moreover, water viscosity measurements were obtained through Sader’s fit with 4% accuracy, in a trace of 10 ms in length at 1 MHz acquisition frequency, which is faster than any other previous data for viscosity assessments. It should be noted that in order to achieve a good definition of the first resonant mode of the cantilever (the value used to set the lower limit of frequency detection), a minimal condition is that the signal extends for at least 10 cycles. In this case, the cantilever’s resonant frequency in water is in the order of KHz, which leads to a minimum of 10 ms acquisition time. In the future, this may facilitate access to the unfolding of two states protein, with a transition time scale of several milliseconds [27, 28], or the assessment of other biomolecule unfolding processes, such as the unfolding of DNA hairpins, with rates within similar ranges [29].

Thus, time resolution is currently an important improvement for micro cantilever techniques with respect to the BTCP methodology whose time resolution is limited by the number of trajectories required for the analysis of Brownian’s motion. According to Tu’s work [6], this analysis can be achieved in about 60*s* per viscosity point, although it is claimed that BTCP has the potential to improve time resolution through better images analysis and high speed imaging. Indeed, a complete discussion on the typical viscometric methods used in protein research, including their volume requirements, is given by Josephson et al [5]. In particular, recent improvements in particle tracking microrheology of proteins [5] indicate that the sample volume could be decreased down to 2*μ*l, with an acquisition time per data point of about 2s and uncertainty less than 2%.

Thus, it seems clear that the so-called “bottleneck” in kinetics studies is due to the high inertia of experimental devices in response to sudden changes in the protein environment. Indeed, switching denaturant concentration in a protein solution may require relatively long delays before reaching thermalization, homogenization of concentration fields, or the damping of mechanical perturbations induced during these processes. In the future, devices inspired by microfluids [5] may help to prevent these undesirable effects, allowing for reliable and time resolved viscosity measurements.

## Conclusion

Accurate determination of the viscosity of a protein solution, even at minute solution volume, was achieved through a fitting procedure based on the Sader’s model for the PSD of the cantilever’s fluctuations. Increases in the solution’s viscosity under denaturant action are described with the assumption that proteins are hard spheres with a hydrodynamic radius that increases during unfolding, and modeled accordingly using the Einstein-Batchelor’s formula. Via the direct measurement of the hydrodynamic radius of proteins, obtained using dynamic light scattering, it is shown that predictions of Einstein-Batchelor’s formula are verified in the BSA protein. Thus, conclusions are drawn that this methodology proved to be reliable for the study of protein folding in the microliter-sized samples, at vanishing shear stress, achieving an improved time resolution.

## Supporting information

S1 File Theoretical and experimental basis for the viscosity determination. 1: Mathematical basis of cantilever fluctuations. 2.1: Determination of viscosity through the tracking of the resonance frequency of the cantilever. 2.2: Viscosity determination through the fit of the experimental PSD to the theoretical PSD of Sader’s model. 3: Correction to the hydrodynamic radius due to the apparent diffusion in DLS measurements.
(PDF)

S1 Dataset Several records of cantilever deflection versus time, together with all the Matlab functions necessary to obtain the cantilever geometrical parameters. (ZIP)

S2 Dataset Examples of cantilever deflection versus time together with the Matlab functions necessary for obtaining the viscosity and density of the solution. (ZIP)

S1 Fig Fit of viscosity of urea solution (Eq 6) as a function of resonance frequency (*ω*_*r*_) for the calibration of *c*_1_ and *c*_2_. (TIF)

S2 Fig Flux diagram to obtain the geometrical parameters of the cantilever. Blocks indicate the routine names developed for calculation and data handling. These routines are available in the S1 and S2 Dataset.
(TIF)

S3 Fig Fit of cantilever parameters. A) Interactive windows to fit the geometrical parameters of the cantilever. Maximum and minimum values of geometrical parameters are inputs. The exact position, x, is adjusted to place the detecting laser on the cantilever. The *α*_*m*_ parameter is used for coated cantilevers accounting for the added mass. The *f*_*min*_ and *f*_*max*_ terms define the frequency range for PSD fitting. B) A fitting example; green: experimental data, blue: fit to the PSD’s. Background noise is defined by “BGnoise” shown in (A), taken from PSD average at high frequency (above 10^5^ Hz).
(TIF)

S4 Fig Flux diagram for the determination of viscosity and density. (TIF)

S5 Fig Apparent hydrodynamic radius *R*_*app*_ as function of BSA concentration. Blue: folded protein in absence of urea. Red: unfolded state in 7M urea concentration. The best fits of both set of data to Eq 15 are indicated with solid lines.
(TIF)
