# Supplementary material for: Detecting protein folding by thermal fluctuations of microcantilevers
Source: PLoS One. 2017 Dec 21;12(12):e0189979. doi: 10.1371/journal.pone.0189979 (PMC5739453; doi:10.1371/journal.pone.0189979)
Supplement: S1 File — 1: Mathematical basis of cantilever fluctuations. 2.1: Determination of viscosity through the tracking of the resonance frequency of the cantilever. 2.2: Viscosity determination through the fit of the experimental PSD to the theoretical PSD of Sader’s model. 3: Correction to the hydrodynamic radius due to the apparent diffusion in DLS measurements. (PDF) [file pone.0189979.s001.pdf]

Supporting information

1 Mathematical Basis of cantilever fluctuations

The physical model of a fluctuating cantilever in a fluid environment was summarized in [1], we recall here the main results necessary for the reader in order to assess the fluid viscosity. These basis can be found in great detail in the original work of Sader [2] and Bellon [3].

The main quantity is the power spectrum density, $S_{d_n}(\omega)$, for each normal mode of the deflection d for a microcantilever immersed a fluid environment at temperature T. This writes,

$$S_{d_n}(\omega) = \frac{2k_BT}{\pi}\frac{\gamma_{\text{eff}}(\omega)}{(k_n - m_{\text{eff}}(\omega)\omega^2)^2 + (\gamma_{\text{eff}}(\omega)\omega)^2}, \tag{1}$$

where d_n is the amplitude of the $n-$th normal mode of the cantilever and a fluctuating variable due to the thermal bath, $\gamma_{\text{eff}}(\omega)$ is the frequency dependent effective damping coefficient, $m_{\text{eff}}(\omega)$ is the frequency dependent effective mass, k_n is the stiffness of the $n-$th mode, k_B is the Boltzmann constant and ω is the angular frequency. The effective values m_{eff} and γ_{eff} write,

$$m_{\text{eff}}(\omega) = m + m_f\Gamma_r(\omega) \tag{2}$$
$$\gamma_{\text{eff}}(\omega) = m_f\omega\Gamma_i(\omega), \tag{3}$$

where m is the mass of the cantilever, $m_{\text{f}} = \pi\rho LW^2/4$ is the mass of the cylinder of fluid of density ρ surrounding the cantilever of length L and width W and $\Gamma = \Gamma_r + i\Gamma_i$ is the hydrodynamic function in the Sader's model context, whose values are dependent of the fluid density and viscosity. For the sake of completeness, we introduce explicitly the hydrodynamic function for a rectangular beam,

$$\Gamma(\omega) = \Omega(\omega)\Gamma_{\text{circ}}(\omega), \tag{4}$$

where Γ_{circ} is the hydrodynamic function for a cylinder expressed as,

$$\Gamma_{\text{circ}}(\omega) = 1 + \frac{4iK_1(-i\sqrt{i\text{Re}})}{\sqrt{i\text{Re}}K_0(-i\sqrt{i\text{Re}})}, \tag{5}$$

where K_0 and K_1 are modified Bessel functions of the third kind and Re is the Reynolds number, expressed as,

$$\text{Re} = \frac{\rho W^2\omega}{4\eta}, \tag{6}$$

where ρ is the density of the fluid where the cantilever is immersed, W is the width of the cantilever and η is the viscosity of the fluid. $\Omega(\omega) = \Omega_r + i\Omega_i$ is a correction function,

expressed in the real and imaginary parts as,

$$
\begin{aligned}
\Omega_r(\omega) &= (0.91324 - 0.48274r + 0.46842r^2 - 0.12886r^3 \\
&+ 0.044055r^4 - 0.0035117r^5 + 0.00069085r^6) \\
&\times (1 - 0.56964r + 0.48690r^2 - 0.13444r^3 \\
&+ 0.045155r^4 - 0.0035862r^5 \\
&+ 0.00069085r^6)^{-1} \qquad (7) \\
\Omega_i(\omega) &= (-0.024134 - 0.029256r + 0.016294r^2 \\
&- 0.00010961r^3 + 0.000064577r^4 \\
&- 0.000044510r^5) \times (1 - 0.59702r + 0.55182r^2 \\
&- 0.18357r^3 + 0.079156r^4 - 0.014369r^5 \\
&+ 0.0028361r^6)^{-1} \qquad (8) \\
r &= \log_{10}(\mathrm{Re}). \qquad (9)
\end{aligned}
$$

The physical origin of the hydrodynamic function can be found in Sader's work [2]. It is worth nothing that in Sader's model the dissipation is assumed homogeneous along the cantilever. If in addition, the thermal noise is uncorrelated on different normal modes [3], the PSD of the deflection d is written as,

$$S_d(x,\omega) = \sum_{n=1}^{\infty} S_{d_n}(\omega) \left|\phi_n(x)\right|^2, \qquad (10)$$

where ϕ_n are the eigenfunctions of the Euler Bernoulli equation for a cantilever and x is the spatial coordinate along the cantilever length, indicating here the position of the laser spot, by means which deflection is measured. These eigenfunctions write,

$$\phi_n(x) = \left[\cos\left(\alpha_n \frac{x}{L}\right) - \cosh\left(\alpha_n \frac{x}{L}\right)\right] - \frac{\cos\alpha_n + \cosh\alpha_n}{\sin\alpha_n + \sinh\alpha_n}\left[\sin\left(\alpha_n \frac{x}{L}\right) - \sinh\left(\alpha_n \frac{x}{L}\right)\right], \qquad (11)$$

where α_n are the eigenvalues, which in the absence of external forces are the solutions of $1 + \cos\alpha_n \cosh\alpha_n = 0$. The first eigenvalues are: $\alpha_1 = 1.875$, $\alpha_2 = 4.695$, $\alpha_3 = 7.855$ and approximate to $\alpha_n = (n - 1/2)\pi$ for higher n.

2 Data analysis

2.1 Determination of viscosity by tracking of the resonance frequency of the cantilever

The use of the Eq 6 in the main text leads to the determination of viscosity of the solution through the changes in the resonance frequency, ω_r of the cantilever. A series of steps allows us to find the viscosity from the PSD:

- A set of data taken around the maximum of the experimental PSD is fitted to a Gaussian distribution and the resonance frequency, f_r, is thus obtained from the Gaussian maximum. The mean value $\overline{f_r}$ is obtained from the average of $N = 20$ data.
  The vacuum resonance frequency, f_0, is obtained in air in the same manner.

This brings the mean value of the resonance frequency and the respective error, calculated as the standard deviation, $f_r = \overline{f_r} + \Delta f_r$. The angular frequency is calculated as usually, $\overline{\omega_r} = 2\pi\overline{f_r}$, $\Delta\omega_r = 2\pi\Delta f_r$.

- In order to quantify the error propagation in the Eq 6 (see the main text), the parenthesis is distributed to obtain a expression for η, $\eta = A + B + C$. Each term in viscosity is analyzed :

$$
\begin{aligned}
A &= \frac{c_1 \cdot \omega_0^4}{\omega_r^3} \\
\Delta A &= \sqrt{\left(\frac{\omega_0^4 \Delta c_1}{\omega_r^3}\right)^2 + \left(\frac{4c_1\omega_0^3\Delta\omega_0}{\omega_r^3}\right)^2 + \left(\frac{3c_1\omega_0^4\Delta\omega_r}{\omega_r^4}\right)^2} \\
B &= \frac{2 \cdot c_1 \cdot (c_2+1)\omega_0^2}{\omega_r} \\
\Delta B &= \sqrt{\left(\frac{2(c_2+1)\omega_0^2\Delta c_1}{\omega_r}\right)^2 + \left(\frac{2c_1\omega_0^2\Delta c_2}{\omega_r}\right)^2 + \left(\frac{4c_1(c_2+1)\omega_0\Delta\omega_0}{\omega_r}\right)^2 + \left(\frac{2c_1(c_2+1)\omega_0^2\Delta\omega_r}{\omega_r^2}\right)^2} \\
C &= c_1 \cdot (c_2+1)^2\omega_r \\
\Delta B &= \sqrt{((c_2+1)^2\omega_r\Delta c_1)^2 + (2c_1(c_2+1)\omega_r\Delta c_2)^2 + (c_1(c_2+1)^2\Delta\omega_r)^2} \\
\eta &= A + B + C \\
\Delta\eta &= \Delta A + \Delta B + \Delta C
\end{aligned}
$$

- The parameters c_1 and c_2 depends on the cantilever geometry. These two parameters must be found through a calibration procedure that is based on the nominal values of the urea viscosity, η_s, [4] and the fit of the experimental data to the Eq 6. The results are shown in S1 Fig and the respective values are: $c_1 = (1.0 \pm 0.3) \cdot 10^{-10}$ and $c_2 = 0.39 \pm 0.14$.

2.2 Viscosity determination through the fit of the experimental PSD to the theoretical PSD of Sader's model.

2.2.1 Geometric parameters of the cantilever.

The data analysis includes two main steps; first the geometry of the cantilever is determined through a calibration procedure in a known fluid (water), and second the density and viscosity of each sample are determined. The analysis is done through codes developed in Matlab language, available in S1 Dataset for geometry determination and S2 Dataset for density and viscosity.

The approximative geometrical properties of cantilever and its stiffness are known. However, a precise knowledge of these quantities is crucial for the accuracy of our method. We therefore perform a fine tuning of these parameters by registering the PSDs of cantilever when it is immersed in ultra pure water, whose density and viscosity are known for a given temperature. The geometrical parameters, L, W, e and k, are then tuned until the calculated PSD accurately reproduce the experimental PSD. The procedure is summarized through the diagram S2 Fig, which indicates the main calculation routines. The average of the power spectrum density $< PSD >_e$ is obtained from 20 acquisitions. Initial values of

cantilever geometry loaded together with the exact values of the viscosity and density of the reference fluid. For a set of geometrical parameters, a trial form of the power spectrum density $< PSD >_t$ is obtained using the specified routines. An iterative process runs until the difference $< PSD >_e - < PSD >_t$ is minimized in a frequency interval containing the desire frequency bandwidth. The whole process is summarized in a master routine whose main panel is given, an example of fitting of the PSD is also given (see S3 Fig).

2.2.2 Determination of the viscosity and density.

The procedure to determine an unknown fluid viscosity is carried out in a similar way but the cantilever's geometry and stiffness, optimized previously, are kept fixed while the viscosity and density are free parameters for the fitting procedure. The S4 Fig shows a diagram for the viscosity and density determination, matlab's routines are indicated and available in S2 Dataset.

3 Correction to the hydrodynamic radius due to the apparent diffusion in DLS measurements.

The concentration dependence of the apparent diffusion coefficient gives information about proteins interactions. The effect of the protein concentration is in general considered by extrapolating the value of the apparent diffusion coefficient to zero concentration using [5],

$$D_{app} = D_0(1 + k_D c_{BSA}), \tag{12}$$

where D_{app} is the apparent diffusion coefficient, D_0 is the diffusion coefficient extrapolated to zero concentration, c_{BSA} is the BSA concentration and k_D is the dynamic virial coefficient. The parameter k_D can be related to the thermodynamic properties of the polymer/solvent mixture, depending therefore on the protein and solvent nature.
An important issue is the scaling of the apparent hydrodynamic radius, R_{app} extrapolated to zero concentration, R_0. R_0 is calculated from the diffusion coefficient in the limit of zero BSA concentration, D_0, and given by the Stokes–Einstein relation,

$$R_0 = \frac{k_B T}{6\pi\eta_s D_0}, \tag{13}$$

where η_s is the solvent viscosity. If the apparent hydrodynamic radius, R_{app} is calculated using the apparent diffusion coefficient D_{app} and the solvent viscosity, the expression relating the hydrodynamic radius reads,

$$R_{app} = \frac{R_0}{(1 + k_D c_{BSA})} \tag{14}$$

Thus, through measurements of the hydrodynamic radius from DLS, for increasing BSA concentrations and for fixed buffer and denaturant concentration, the hydrodynamics radius R_0 and the k_D constant can be obtained, allowing for a direct comparison with hydrodynamic radius from viscosity measurements.

In fact, in S5 Fig, for increasing BSA concentrations, while keeping constant denaturant concentration, a small decrease in the mean apparent radius is observed. This effect is more pronounced for higher denaturant concentrations. For BSA in buffer, we obtained $k_{Df} = 0.17 \pm 0.08$ (mM)$^{-1}$, which is in agreement with the value reported by Medda et al under

similar conditions [6], and for BSA in urea at concentration 7M, our measurement provide $k_{Duf} = 0.60 \pm 0.27$ (mM)$^{-1}$. Finally, in order to estimate the corrected hydrodynamic radius for all urea concentrations, a linear interpolation for the k_D coefficient was assumed. The corrected hydrodynamic radius for DLS are then contrasted to the corresponding protein radius obtained from viscosity assessment (Fig 4B in the manuscript).

References

[1] Aguilar Sandoval F, Sepúlveda M, Bellon L, Melo F. High Resolution Viscosity Measurement by Thermal Noise Detection. Sensors. 2015;15(11):27905–27916.

[2] Sader JE. Frequency response of cantilever beams immersed in viscous fluids with applications to the atomic force microscope. J Appl Phys. 1998;84(1):64–76.

[3] Bellon L. Thermal noise of microcantilevers in viscous fluids J Appl Phys. 2008; 104(10):104906.

[4] Kawahara K, Tanford C. Viscosity and density of aqueous solutions of urea and guanidine hydrochloride. J Biol Chem. 1966;241(13):3228–3232.

[5] Adel A, Nadia M, Mohamed O, Abdelhafidh G. Study of thermally and chemically unfolded conformations of bovine serum albumin by means of dynamic light scattering. Mater Sci Eng C. 2008;28(5):594–600.

[6] Medda L, Monduzzi M, Salis A. The molecular motion of bovine serum albumin under physiological conditions is ion specific. Chem Commun. 2015;51(30):6663–6666.
