# Supplementary figures and images for: Detecting protein folding by thermal fluctuations of microcantilevers
Source: PLoS One. 2017 Dec 21;12(12):e0189979. doi: 10.1371/journal.pone.0189979 (PMC5739453; doi:10.1371/journal.pone.0189979)

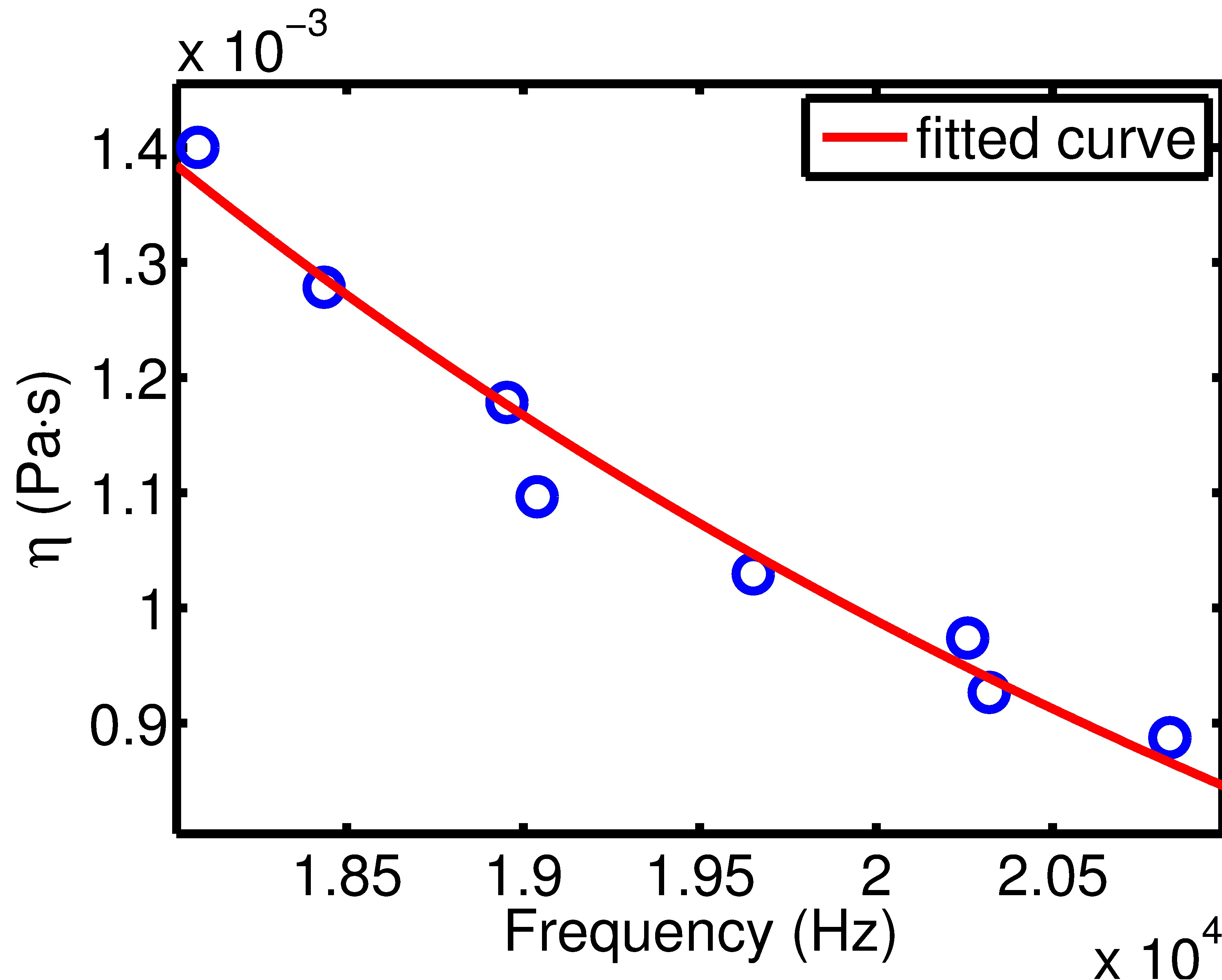

Supplement: S1 Fig — (TIF) [file pone.0189979.s004.tif]

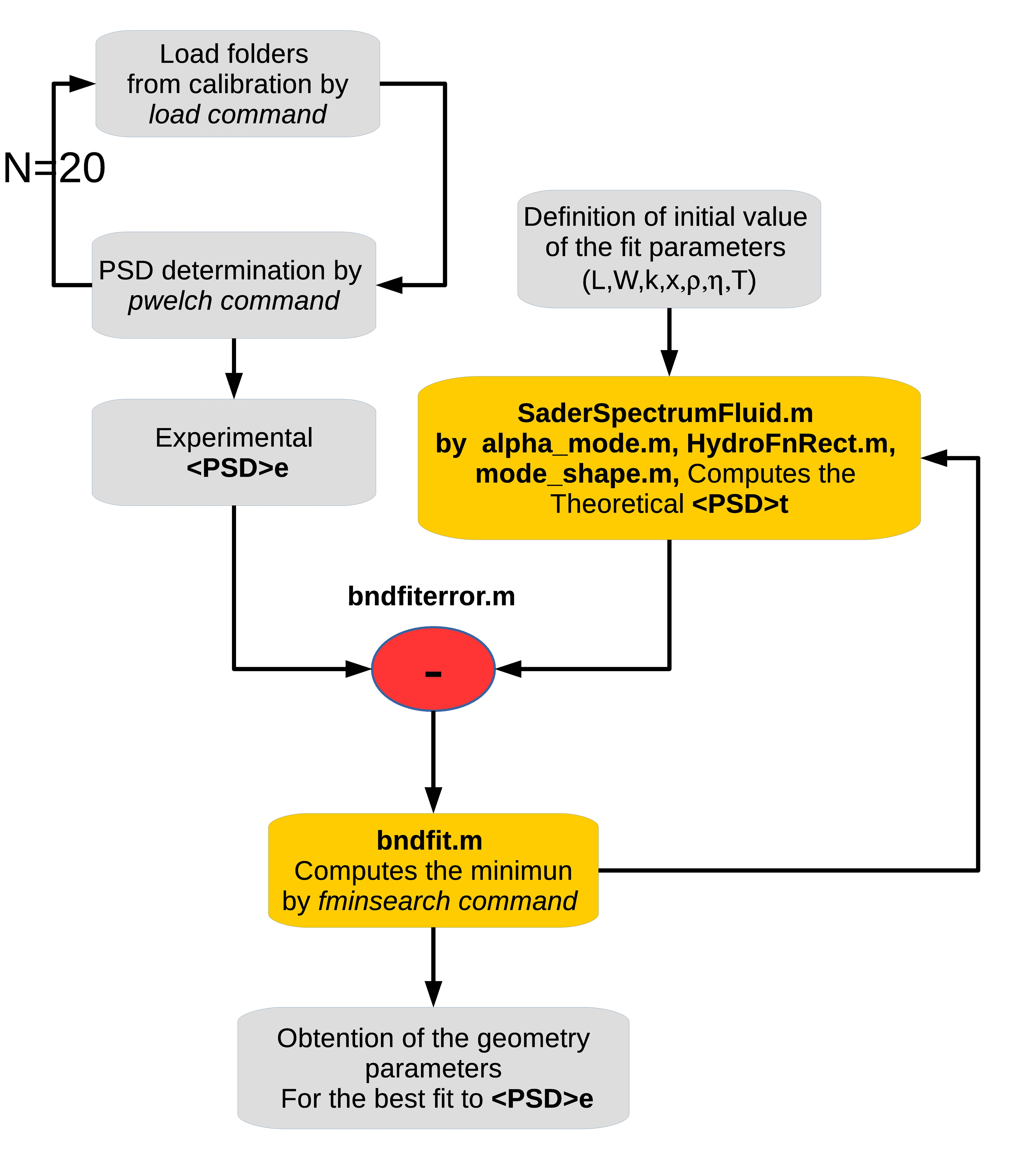

Supplement: S2 Fig — Blocks indicate the routine names developed for calculation and data handling. These routines are available in the S1 and S2 Dataset. (TIF) [file pone.0189979.s005.tif]

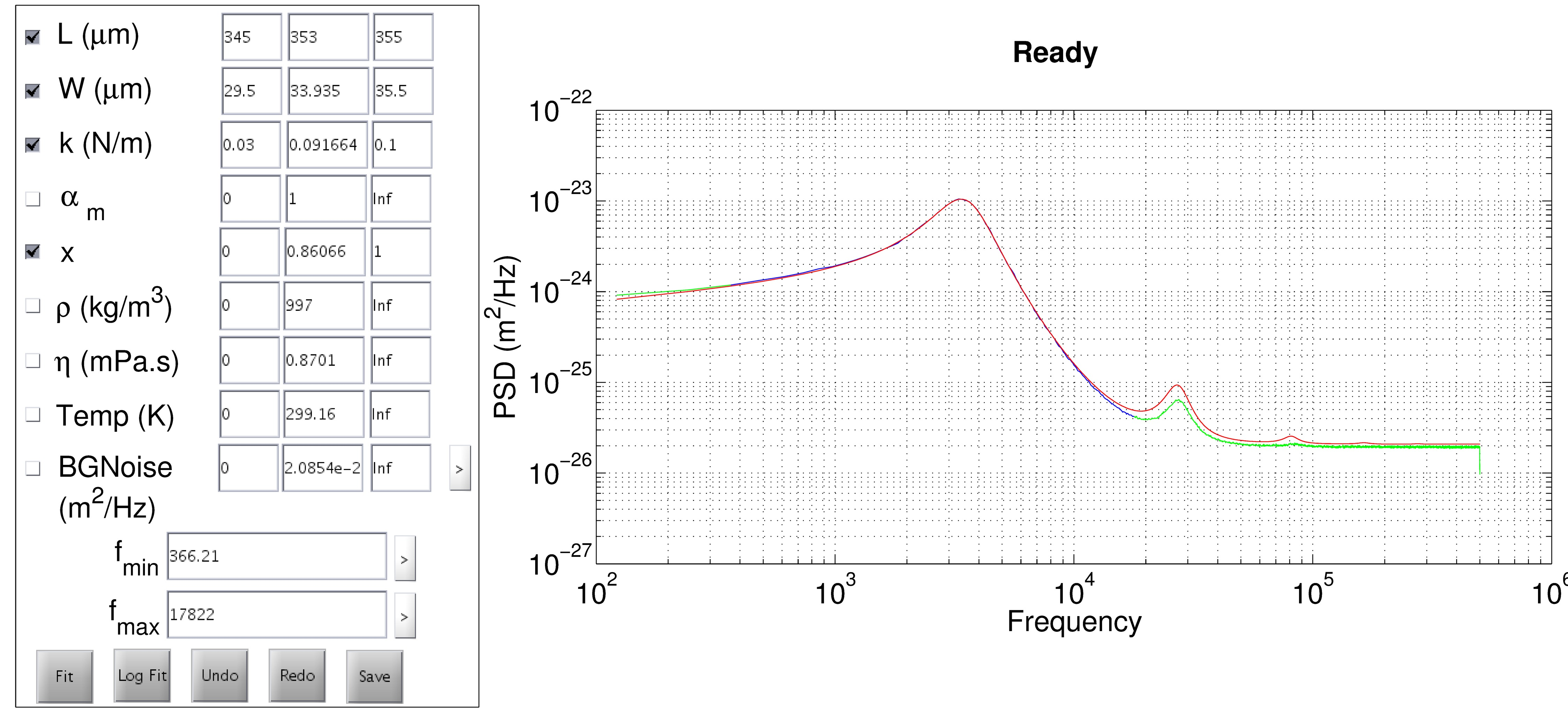

Supplement: S3 Fig — A) Interactive windows to fit the geometrical parameters of the cantilever. Maximum and minimum values of geometrical parameters are inputs. The exact position, x, is adjusted to place the detecting laser on the cantilever. The αm parameter is used for coated cantilevers accounting for the added mass. The fmin and fmax terms define the frequency range for PSD fitting. B) A fitting example; green: experimental data, blue: fit to the PSD’s. Background noise is defined by “BGnoise” shown in (A), taken from PSD average at high frequency (above 105 Hz). (TIF) [file pone.0189979.s006.tif]

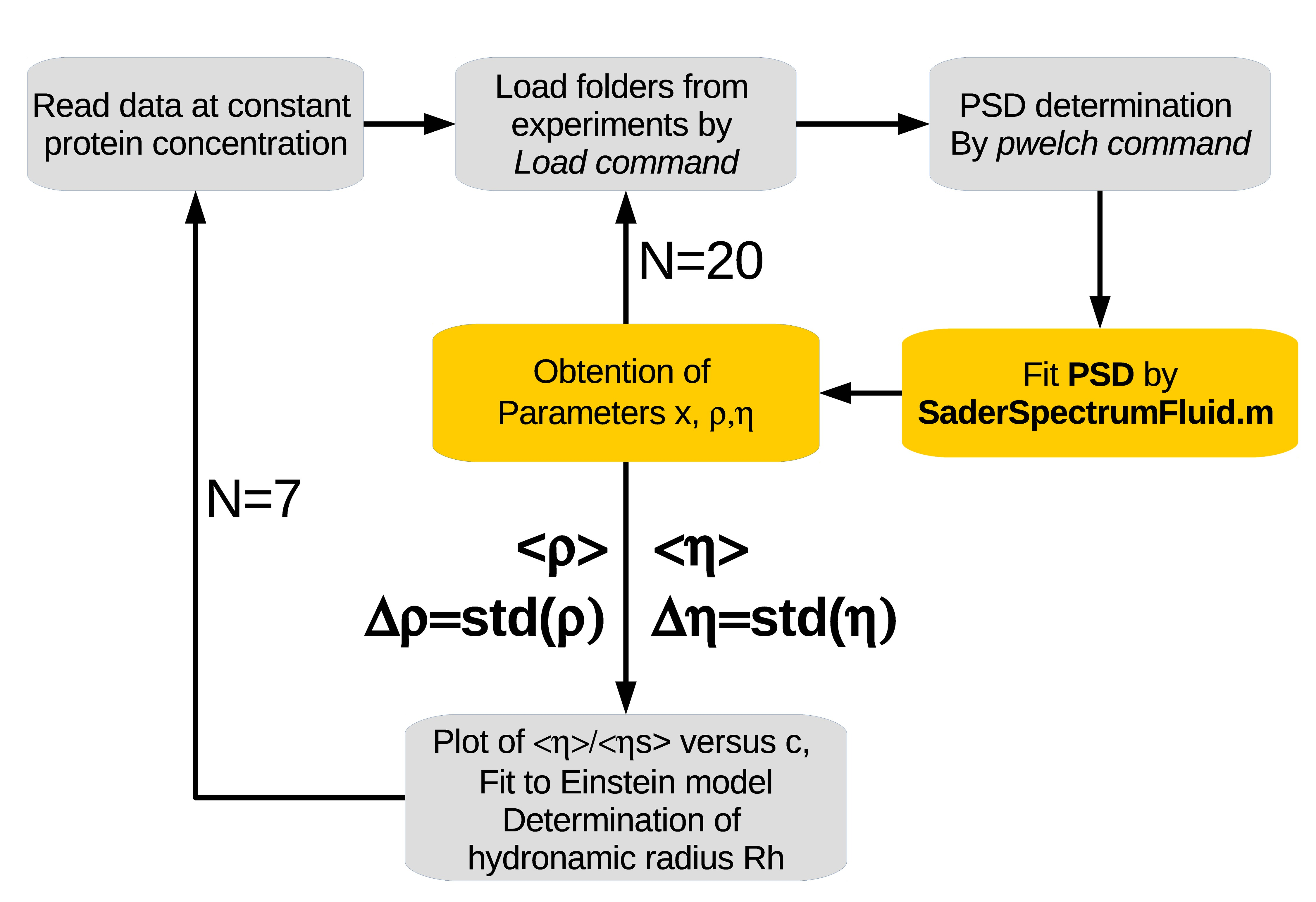

Supplement: S4 Fig — (TIF) [file pone.0189979.s007.tif]

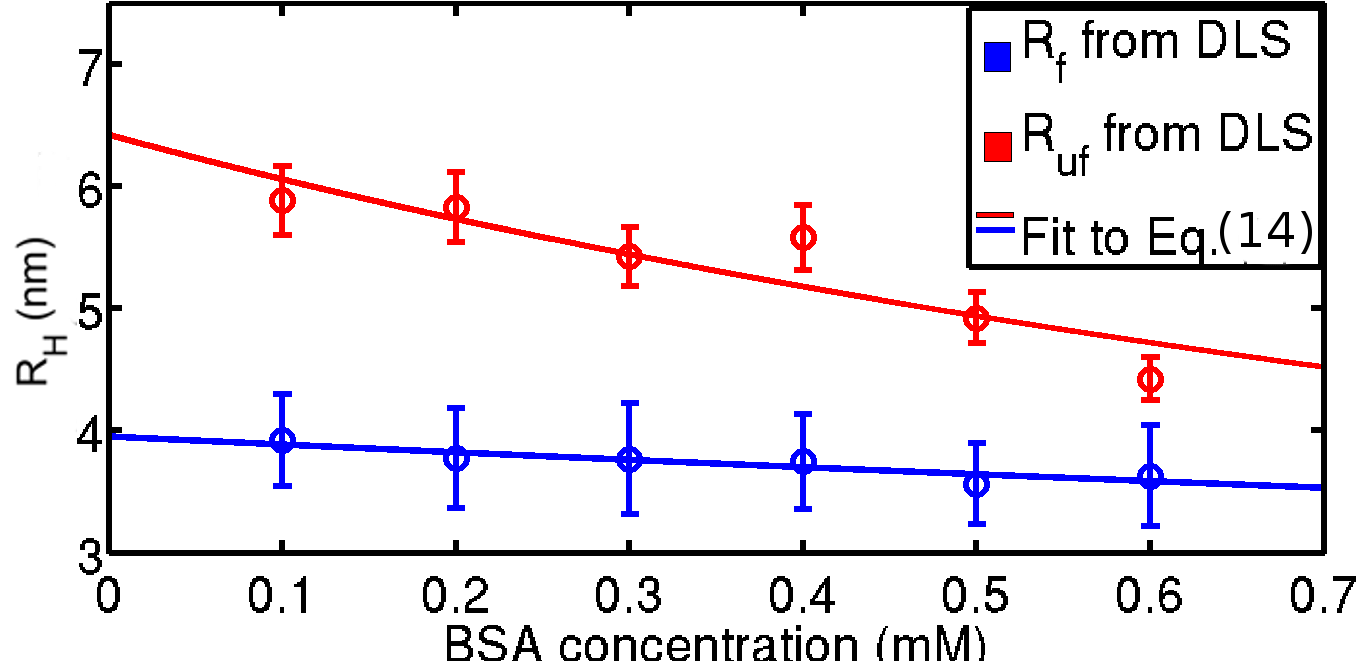

Supplement: S5 Fig — Blue: folded protein in absence of urea. Red: unfolded state in 7M urea concentration. The best fits of both set of data to Eq 15 are indicated with solid lines. (TIF) [file pone.0189979.s008.tif]
